# Conserved Mosquito/Parasite Interactions Affect Development of *Plasmodium falciparum* in Africa

**DOI:** 10.1371/journal.ppat.1000069

**Published:** 2008-05-16

**Authors:** Antonio M. Mendes, Timm Schlegelmilch, Anna Cohuet, Parfait Awono-Ambene, Maria De Iorio, Didier Fontenille, Isabelle Morlais, George K. Christophides, Fotis C. Kafatos, Dina Vlachou

**Affiliations:** 1 Imperial College London, Division of Cell and Molecular Biology, Faculty of Natural Sciences, South Kensington Campus, London, United Kingdom; 2 Institut de Recherche pour le Développement - Laboratoire de Lutte contre les Insectes Nuisibles, UR 016, BP 64501, Montpellier, France; 3 Organisation de Coordination de la lutte contre les Endémies en Afrique Centrale, Laboratoire de Recherche sur le Paludisme, BP 288, Yaoundé, Cameroon; 4 Imperial College London, Division of Epidemiology, Department of Public Health and Primary Care, Faculty of Medicine, St Mary's Campus, London, United Kingdom; Stanford University, United States of America

## Abstract

In much of sub-Saharan Africa, the mosquito *Anopheles gambiae* is the main vector of the major human malaria parasite, *Plasmodium falciparum*. Convenient laboratory studies have identified mosquito genes that affect positively or negatively the developmental cycle of the model rodent parasite, *P. berghei*. Here, we use transcription profiling and reverse genetics to explore whether five disparate mosquito gene regulators of *P. berghei* development are also pertinent to *A. gambiae/P. falciparum* interactions in semi-natural conditions, using field isolates of this parasite and geographically related mosquitoes. We detected broadly similar albeit not identical transcriptional responses of these genes to the two parasite species. Gene silencing established that two genes affect similarly both parasites: infections are hindered by the intracellular local activator of actin cytoskeleton dynamics, WASP, but promoted by the hemolymph lipid transporter, ApoII/I. Since *P. berghei* is not a natural parasite of *A. gambiae*, these data suggest that the effects of these genes have not been drastically altered by constant interaction and co-evolution of *A. gambiae* and *P. falciparum*; this conclusion allowed us to investigate further the mode of action of these two genes in the laboratory model system using a suite of genetic tools and infection assays. We showed that both genes act at the level of midgut invasion during the parasite's developmental transition from ookinete to oocyst. ApoII/I also affects the early stages of oocyst development. These are the first mosquito genes whose significant effects on *P. falciparum* field isolates have been established by direct experimentation. Importantly, they validate for semi-field human malaria transmission the concept of parasite antagonists and agonists.

## Author Summary

Malaria is a parasitic infectious disease transmitted by mosquitoes. It impacts half the population of the world and kills 1 to 3 million people every year, the vast majority of whom are children aged below 5 in sub-Saharan Africa. There, the deadliest parasite is *Plasmodium falciparum* and its most important vector is the mosquito *Anopheles gambiae*. This study identifies for the first time specific *A. gambiae* genes that demonstrably regulate the density of mosquito infection by *P. falciparum* parasites circulating in malaria patients in Africa. These genes function in mosquito lipid transport and intracellular actin cytoskeleton dynamics, and act as an agonist and an antagonist, respectively, of the parasite ookinete-to-oocyst developmental transition. Importantly, our study validates for *P. falciparum* the concept of mosquito genes that support or hinder parasite development, a concept that we defined previously using a laboratory model system. Thus, the work constitutes a major contribution to understanding meaningful mosquito/parasite interactions in natural transmission conditions.

## Introduction

Sub-Saharan Africa is the major and persistent focus of malaria, one of the most devastating scourges of humankind. There, *P. falciparum* is by far the most important human malaria parasite and *A. gambiae* its most important vector. Laboratory infections of *A. gambiae* by the convenient rodent model parasite, *P. berghei,* elicit broad responses encompassing multiple mosquito genes; some belong to classical innate immunity and act systemically, while others participate in local epithelial responses [1]–[6]. Extensive laboratory experiments with this parasite have established that the outcome of infection depends on finely balanced factors that affect, positively or negatively, the developmental cycle in the mosquito, mostly at the bottleneck of invading the midgut epithelium. However, to identify molecular interactions potentially suitable for developing novel interventions to interrupt the human malaria cycle, it is now important to analyze the invasion response and its consequences in more natural settings. Here, we analyze invasion responses and their consequences, using endemic populations of *P. falciparum*, the human malaria parasite, and a strain of *A. gambiae* established from mosquitoes collected in the same area.

The importance of field-based analysis of malaria transmission is underlined by recent studies of vector/parasite interactions. *Anopheles* mosquitoes are largely inhospitable to *Plasmodium* [7], and most species host few, if any, *Plasmodium* species in nature. Only a small number of *Anopheles*/*Plasmodium* species combinations have evolved to support effectively this parasitism. Even these *Anopheles* vectors eliminate most of the input parasites. Further, the level of *A. gambiae* resistance to *P. falciparum* apparently depends on specific genotype*genotype interactions: certain mosquitoes resist one subset of parasite genotypes while others resist a different subset [8]. Therefore, effective transmission may require specific compatibility between vector and parasite genotypes; conclusions from a particular combination may not apply to other combinations. Consistent with this concept, several different, operationally defined mosquito quantitative trait loci (QTLs) are associated with resistance against specific parasite species or genotypes [9],[10]. For instance, the genetically selected *A. gambiae* L3-5 strain possesses genetic traits that confer refractoriness to the simian *P. cynomolgi* parasite and numerous other species of *Plasmodium*, including non-African *P. falciparum* isolates, but not (or only very poorly) to its sympatric African *P. falciparum* isolates [11]. This observation suggests that sympatric mosquito/parasite populations may have co-evolved in permitting significant transmission intensities in the field. Further, the *A. gambiae LRIM1* gene promotes and *CTL4* inhibits *P. berghei* lysis and melanization [4]; the same LRIM1/CTL4 module apparently does not affect the outcome of sympatric *A. gambiae*/*P. falciparum* infections [12]. Such data suggest that vector immunity may have been co-adapted during co-evolution with the parasite [10],[13]. Nonetheless, *P. falciparum* clearance and melanization have been observed in infected wild *A. gambiae* in Africa, and thus constitute low frequency but natural phenotypes, in sympatric combinations [14],[15]. Mosquito loci that regulate the infection load or parasite melanization in the field have been mapped [10],[14] further suggesting that resistance is a default outcome of infection but is compromised in sympatric interactions.

Major cytoskeletal reorganization is a predominant response of parasite-invaded midgut cells [16],[17], and is accompanied by transcriptional regulation of genes implicated in cytoskeletal dynamics [5]. For example the gene encoding WASP, a local activator of actin cytoskeleton reorganization, is upregulated in the midgut epithelium during *P. berghei* invasion, and its silencing significantly increases *P. berghei* infection loads [5]. In contrast, *P. berghei* upregulates the mosquito precursor of Apolipophorin II/I (ApoII/I), a key circulating lipid transport regulator which appears to benefit both vector and parasite: its silencing disrupts mosquito egg development and drastically decreases parasite oocyst numbers [5].

Here, we examined whether local epithelial and systemic but not-classical immune responses of *A. gambiae* are pertinent to interactions between field isolates of *P. falciparum* and mosquitoes of a strain derived from a sympatric *A. gambiae* population. We discovered that in this strain both *WASP* and *ApoII/I* respond transcriptionally to *P. falciparum* as they do to *P. berghei*, and that their silencing impacts development of both parasites in the same direction. The conservation of these mosquito reactions against two distantly related parasite species allowed us to investigate the mechanism of action of these two genes in the tractable laboratory model setting. Using as tools *P. berghei* and *A. gambiae* strains that are either genetically selected (L35) or epigenetically modified (*CTL4* kd), we clearly demonstrate that ApoII/I facilitates ookinete invasion of the mosquito midgut as well as development of early oocysts, while WASP acts only at the level of ookinete invasion. Evidently, WASP-mediated actin reorganization in the invaded epithelium is detrimental to both parasites whereas lipid transport by ApoII/I is beneficial, to both mosquito egg and *Plasmodium* development, in human as well as rodent malaria infections. Thus, despite the existence of genotype*genotype specific interactions [8], some important aspects of mosquito/parasite interactions are evolutionarily conserved. Further, the model laboratory transmission system can provide leads concerning genetic regulators; these must then be validated by translational research in more demanding, field-based systems of human malaria transmission.

## Results

### Selection of candidate genes for expression profiling and silencing

Four *A. gambiae* genes representing diverse systemic or local epithelial responses elicited by *P. berghei* infections were selected for assessing in well-controlled experiments their involvement in transcriptional responses to infection by sympatric *P. falciparum* isolates and their RNAi-mediated silencing effect on parasite infectivity. As described in the introduction, *ApoII/I* and *WASP* are transcriptionally induced by *P. berghei* but have opposite effects on parasite development in the vector [5]. Two other genes were also identified as being transcriptionally induced during infection, but, their silencing had no effect on parasite infectivity [5]: *CATHB* encodes the proapoptotic enzyme Cathepsin B and was tested in light of observed apoptosis of parasite-invaded cells [16],[17], while *KIN1* encodes a histidine-rich putative antimicrobial peptide produced under NF-κB control [18]. *KIN1* is transcriptionally induced by bacteria and *P. falciparum* laboratory strain infections [2],[19], suggesting involvement in immunity. Finally, we tested a fifth gene, *ApoIII,* which encodes a polypeptide known to combine with ApoI and ApoII to form the insect lipophorin [20] and thus may also be involved in a systemic response to *Plasmodium*.

### *A. gambiae* infections with *P. falciparum* in Africa

*P. falciparum* isolates were sampled during two high malaria transmission seasons, May 2005 and 2006, in a parasitological survey of 3,081 primary school, 5–11 year old, pupils. The survey was conducted in Mfou, a town 30 km outside Yaoundé, Cameroon. It identified an average of 51% *P. falciparum* infection prevalence and 5.9% gametocyte prevalence (% of blood samples with detectable asexual parasite blood stages (ABS) or gametocytes, respectively; Table S1).

Mosquitoes used in this study were from the Yaoundé strain that was colonized in the Yaoundé area in 1988 [21]. A previous study had demonstrated strong loss of polymorphisms and considerable divergence from natural populations in a 20-year old laboratory colony of a different mosquito, the neotropical species *A. albimanus* [22]. We examined to what extend the Yaoundé strain mosquitoes are representative of local populations, by determining the respective genetic diversities and calculating their genetic distance (divergence) from field-collected local *A. gambiae*. The 4ar/r colony of the same species was used as an external reference [23].

We analyzed Single Nucleotide Polymorphisms (SNPs) in 10 immune-related genes spread across the *A. gambiae* genome (Table S2). The results showed that the mean nucleotide diversity (π) in the Yaoundé strain (π = 0.0081) was only slightly lower than in field mosquitoes (π = 0.0095), and that the difference in diversity across loci was not significant (Mann-Withney U test, P>0.57). In contrast, the 4ar/r strain revealed extremely limited mean diversity (π = 0.0005), significantly lower than the diversity of field collected mosquitoes (P<0.001). The divergence between field and Yaoundé mosquitoes was also significant (P<0.001) but relatively low (Fst = 0.1202, D_a_ = 0.0016); the divergence between 4ar/r and field mosquitoes was almost 4-fold more pronounced (Fst = 0.4697, D_a_ = 0.0055, P<0.001). Therefore, despite some differences, the mosquitoes used in our study are a reasonable approximation of the local *A. gambiae* population.

Blood samples were donated by 23 naturally infected gametocyte-carrier volunteers and used to infect Yaoundé mosquitoes in a series of experiments as explained below. In the first experiment we assayed whether the gametocyte density in the blood (gametocytaemia) affects the density of mosquito infection, and thus the subsequent gene expression profiles and our gene silencing experiments. Three to five-day old female mosquitoes were allowed to feed via a membrane on infected blood. Non-blood-fed mosquitoes were removed 24h later, and the mean oocyst density (oocysts per midgut) and infection prevalence (% midguts exhibiting at least one oocyst) were determined 8–10 days post infection (Table 1). Gametocytaemia could not be confirmed in two of the experiments (infections 1 and 18; N/C in Table 1), which were thus excluded from the analysis. To investigate the relationship between gametocyte and oocyst densities we fitted a linear model using oocyst density as the response variable and gametocyte density as the explanatory variable. The oocyst density was log-transformed so that its distribution would better resemble a normal distribution. Indeed, a significant slope coefficient (P<0.05) for the gametocyte density was detected, revealing a correlation between input gametocyte and output oocyst numbers. However, residual analysis revealed that the fit of the model was suboptimal (R^2^ = 0.029); further investigation of the relationship between the two variables would be interesting but is beyond the scope of this study.

**Table 1:** Mosquito infections with *P. falciparum* gametocyte carriers.

Infection	ABS	Gametocytes (per µl blood)	# of midguts	Oocyst range	Oocysts/Midgut Arithmetic mean	Oocysts/Midgut [Gmean (±dSE)]	Prevalence (%)
1	+	N/C	22	0–17	6.5	4.90 (±0.27)	90.9
2	+++	12	93	0–23	11	6.30 (±0.22)	82.7
3	+	50	12	0–32	9.7	4.68 (±0.78)	75
4	+++	166	10	0–99	30.6	9.95 (±1.76)	70
5	-	7	14	0–6	1.57	1.10 (±0.25)	64.5
6	+	10	32	0–11	2.2	1.27 (±0.23)	56.3
7	-	221	32	0–159	34.8	7.06 (±1.21)	56
8	+	14	30	0–7	1.1	0.73 (±0.16)	53
9	+	10	23	0–9	2.1	1.14 (±0.28)	52
10	+	6	35	0–6	1.1	0.71 (±0.14)	48.6
11	+++	36	15	0–15	4.7	1.53 (±0.59)	47
12	-	168	36	0–23	2.8	1.03 (±0.28)	44.4
13	+++	6	61	0–11	1.7	0.7 (±0.16)	36
14	-	107	33	0–12	1.2	0.53 (±0.20)	27.3
15	+	60	31	0–3	0.3	0.20 (±0.08)	19.4
16	+++	88	22	0–2	0.2	0.16 (±0.08)	18
17	-	23	13	0–2	0.5	0.18 (±0.14)	15.4
18	-	N/C	41	0–2	0.2	0.12 (±0.06)	12.2
19	+++	9	19	0–1	0.1	0.04 (±0.04)	5
20	+	12	30	0	0	0	0
21	-	16	20	0	0	0	0
22	+	8	8	0	0	0	0
23	++	11	20	0	0	0	0

Experiments are sorted based on mosquito infection prevalence (% of mosquito midguts showing at least one oocyst). The calculation of gametocytes per µl of blood assumes a standard WBC (White Blood Cell) count of 8000 per µl. ABS shows the density of asexual blood stages (+, 1–50 ABS; ++, 51–500 ABS; +++, 501–5000 ABS). Geometric (Gmean) and arithmetic mean oocyst number per midgut are presented. Midguts from blood-fed mosquitoes lacking oocysts were included in these calculations. Gametocyte presence could not be confirmed for two of the carriers (N/C) on the day of blood sampling.

To investigate the relationship between the gametocyte and mosquito infection prevalence, a logistic regression model was fitted using data obtained from infections 2 to 17 and 19, excluding the two infections with N/C gametocytaemia and no detected oocysts, as shown in Table 1. In this analysis, the mosquito infection prevalence (absence or presence of infection) was used as the response variable and the gametocyte density as the explanatory variable. No significant association was detected between input gametocyte density and infection prevalence (coefficient P = 0.63586).

### Transcriptional responses to *P. falciparum* and *P. berghei* infections

**Figure 1:**
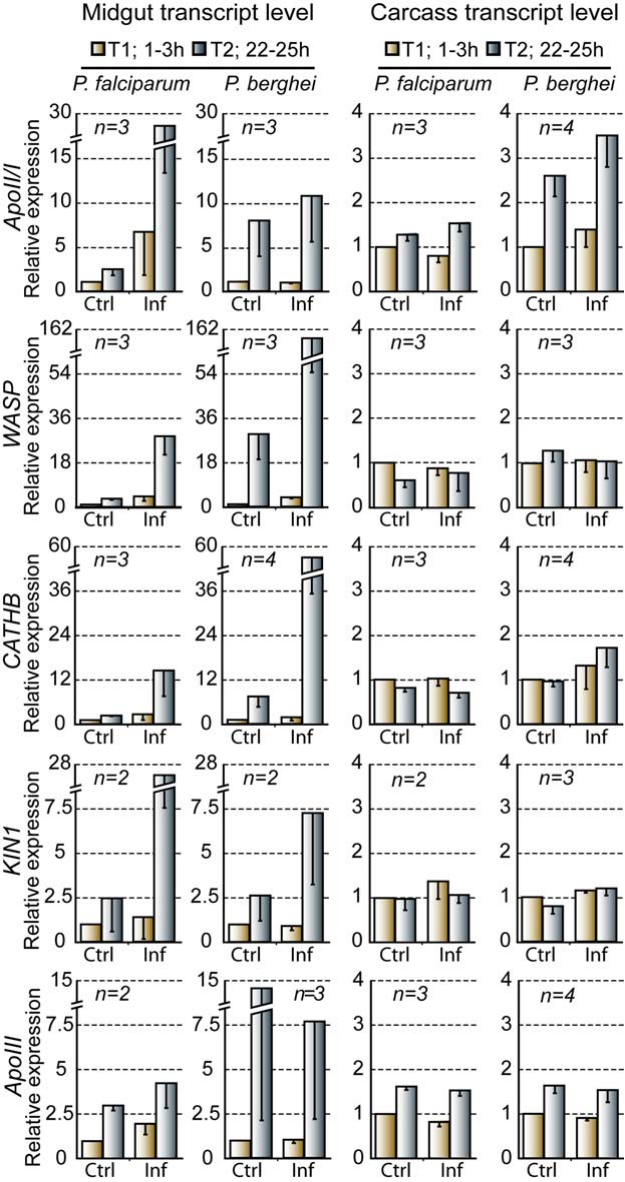
Transcriptional profiles of the five candidate genes. Gene expression assessments in the midgut and carcass of *A. gambiae* mosquitoes (Yaoundé strain) infected (Inf) either with *P. falciparum* natural populations or the *P. berghei* ANKA 2.34 strain, were performed by qRT-PCR at two time periods after bloodmeal: T1 (1–3 h, dark yellow), and T2 (22–25 h, dark blue). Control (Ctrl) *P. falciparum* infections used blood where gametocytes were heat-inactivated, and control (Ctrl) *P. berghei* infections used the non-gametocyte producer strain, ANKA 2.33. The average expression and standard error of biological replicates (n) are shown. Expression data of control samples at T1 were used as a calibrator in each graph.

We used blood donated by the *P. falciparum* gametocyte carriers, to infect mosquitoes and profile the expression of the five candidate genes. Two to three independent biological replicates were performed for each gene; each replicate used a pool of 30–50 mosquitoes and blood from a different carrier. As a control for each replicate, we used 30–50 mosquitoes of the same batch as above, fed on blood originating from the same carrier but depleted of gametocytes by exposure to 42°C. The expression levels of each gene were assessed by quantitative real time RT-PCR (qRT-PCR) in the midgut and carcass (tissues remaining after midgut dissection) at two time periods after blood feeding. T1 (1–3 h) represented the pre-ookinete time period, including gamete fertilization and zygote production in the gut lumen of infected mosquitoes, and T2 (22–25 h) corresponded to ookinete invasion of the midgut epithelium. The replicate results were averaged, and the mean expression levels are presented in Figure 1. In parallel experiments, we used the same design to assess the gene expression levels of the five genes in Yaoundé mosquito infections with *P. berghei*. In this case, the mosquitoes were infected by feeding on mice bearing the ANKA 2.34 strain of *P. berghei*, whereas their respective controls were fed on mice bearing the non-gametocyte producing ANKA 2.33 strain. In the *P. berghei* experiments, the number of replicates ranged from 2 to 4 for different genes (Figure 1). The equivalent designs allowed us directly to compare gene expression between human and rodent malaria infections.

Importantly, with either parasite species, and all candidate genes except *ApoIII* detected higher expression in infected mosquito midguts (especially at T2 when midgut invasion takes place), as compared to their uninfected controls (Figure 1). Despite substantial quantitative variation between biological replicates the trend of induction in infected vs. control samples was highly consistent. This variation between replicates and differences in induction levels between *P. falciparum* and *P. berghei* infections may be due to the differences in infection densities: the geometric mean of oocyst densities were 0.7, 1.1 and 10.0 in the three *P. falciparum* replicate infections, and ranged between 8.6 and 19.8 in the four *P. berghei* infections. *ApoII/I* and *ApoIII* also showed infection-independent temporal induction in the carcass, consistent with the known origin of apolipoproteins in the fat body.

### Effects of gene silencing on *P. falciparum* and *P. berghei* oocyst density

To examine the effect of silencing the five candidate genes on *P. falciparum* infectivity, we used blood from the gametocyte carriers to infect Yaoundé mosquitoes. For each gene, two groups of over 50 freshly emerged adult female mosquitoes taken from the same rearing culture were randomly apportioned to small cages. One, the experimental group, was injected with double-stranded RNA (dsRNA) corresponding to the examined gene and the other (control) group was injected with dsRNA of the *LacZ* gene as described previously [5],[24]. After injection, the two groups were housed identically to eliminate all possible confounding factors. Three to four days later, both groups were allowed to feed via a membrane on the same infected blood source, and the oocyst density was determined at day 8–10 post blood feeding (Table S3). Three to five independent biological replicates were performed for each gene. Each replicate used a different mosquito batch and blood from a different carrier. The silencing efficiency was estimated by qRT-PCR in whole mosquitoes in at least three replicates and averaged; it ranged from 81.4% for *ApoIII* to 51.3% for *CATHB* (Figure S1A). However, as revealed for ApoI and ApoII, silencing is often higher at the protein than at the RNA level (Figure S1B, C).

**Figure 2:**
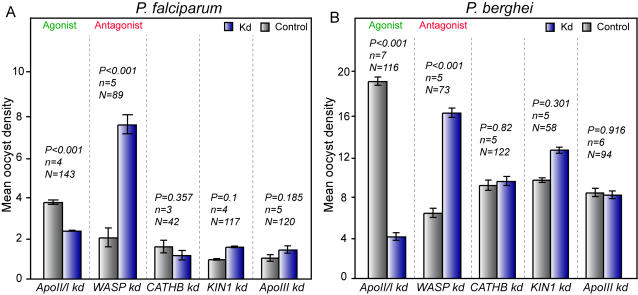
Effect of RNAi gene silencing on *Plasmodium* oocyst densities. Oocyst density in *A. gambiae* midguts infected with *P. falciparum* field isolates (A) and *P. berghei* (B) in paired gene kd (blue columns) and *LacZ* dsRNA-treated controls (grey columns). The geometric means±standard errors of the pooled data from various biological replicate experiments (*n*) are shown. Analysis of data was performed with the Residual Maximum Likelihood (REML) variance components analysis by fitting a mixed effect model. The total number of midguts (N) in each dataset and *P*-values are indicated above each dataset. Silencing of *ApoII/I* and *WASP* affect both the *P. falciparum* and *P. berghei* oocyst densities.

The oocyst density data from all replicates were log-transformed to achieve normality and analyzed by the Residual Maximum Likelihood (REML) variance components analysis by fitting a mixed effect model. In this analysis, we treated the kd-control status as a fixed effect and introduced a random effect for the biological replicate (Figure 2A and Table S3). The difference in infection prevalence between gene kd and control mosquitoes was analyzed using the Chi-square goodness-of-fit test. Relative to their matched controls, *WASP* gene silenced (kd) mosquitoes showed drastic enhancement of *P. falciparum* infection prevalence (80.9% vs. 44.9%; P<0.001) and a highly significant increase in oocyst density (3.7 fold increase; P<0.001). In sharp contrast, the *ApoII/I* kd uniquely decreased the *P. falciparum* oocyst density (−1.6 fold, P<0.001); this silencing also reduced mosquito fitness as it blocked egg development, a phenotype consistent with the proposed function of ApoII/I as the major lipid carrier in the mosquito hemolymph [25]. Silencing the other three genes did not have any significant effect on *P. falciparum* infection prevalence or oocyst density. *ApoIII* silencing also had no effect on mosquito egg development, despite its known involvement, together with ApoII/I, in the insect lipophorin.

A parallel analysis of *P. berghei* infection of Yaoundé mosquitoes showed that silencing *WASP* and *ApoII/I* (but not the other three genes) significantly affects infection loads, in the same direction as for *P. falciparum* (Figure 2B and Table S3). The *WASP* kd strongly increased *P. berghei* oocyst density 2.5-fold (P<0.001), whereas *ApoII/I* kd reduced the density 4.7-fold (P<0.001) and also limited the prevalence, from 85.3% to 61.2% (P<0.001). Earlier, we reported similar results for *P. berghei* in G3 strain mosquitoes [5].

Meta-analysis of the standardized mean difference of the various biological replicates fully corroborated the above results, confirming the agonist nature of ApoII/I and the antagonist nature of WASP, in both *P. falciparum* and *P. berghei* infections. The forest plots for the meta-analysis are shown in Figure S2A.

### ApoII/I facilitates development of *P. berghei* pre-oocyst and early oocyst stages

**Figure 3:**
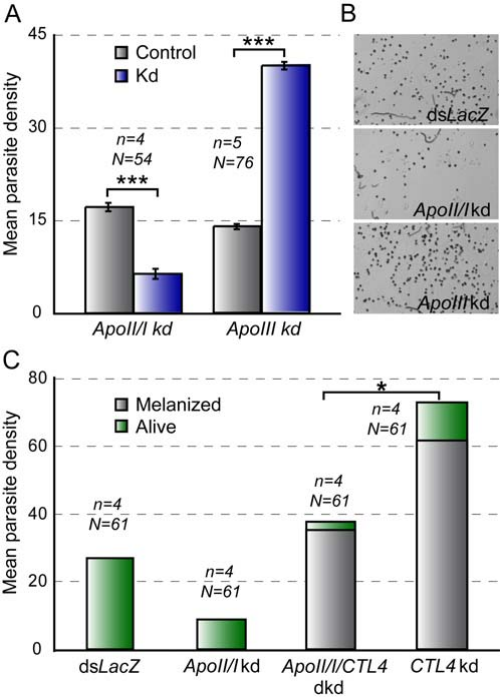
Effects of *ApoII/I* and *ApoIII* silencing on *P. berghei* density in diverse *A. gambiae* genetic backgrounds. (A) Density of melanized ookinetes in the midguts of *A. gambiae* L3-5 mosquitoes. The geometric means±standard errors of the pooled data, in control *LacZ* dsRNA-treated (grey columns) and gene kd (blue columns) from various paired biological replicates (*n*), are shown. Data were analyzed by the REML variance components analysis by fitting a mixed effect model. The total number of midguts (N) in each dataset and *P*-values are indicated. (B) Representative images of L3-5 midguts with melanized parasites in control, *ApoII/I* kd and *ApoIII* kd mosquitoes. Relative to the *dsLacZ* control, melanized parasites are much rarer in the *ApoII/I* kd and much more abundant in the *ApoIII* kd. (C) Epistasis analysis of *ApoII/I* and *CTL4* kds in Yaoundé mosquitoes. Graphs show the arithmetic mean densities of melanized (grey) and alive (green) parasites in the pooled data of four independent replicates calculated for each gene kd, dkd and the *LacZ* control group. Asterisks indicate statistically significant effects of gene kds (*P*<0.05; *, *P*<0.001; ***) as determined by the Residual Maximum Likelihood (REML) variance components analysis.

To infer the parasite stage affected by *ApoII/I* kd, we investigated the effect of this gene on *P. berghei* survival in the parasite-refractory L3-5 strain of *A. gambiae* [11]. L3-5 mosquitoes kill and subsequently melanize or lyse ookinetes as they complete midgut invasion and encounter the hemolymph filtrate in the basal subepithelial space [1],[6]. Therefore, these mosquitoes can be used as a tool to examine the temporal (and spatial) effect of the examined gene against *Plasmodium*: a decrease in the numbers of melanized ookinetes in *ApoII/I*-depleted L3-5 mosquitoes would suggest that *ApoII/I* functions prior to or at the completion of ookinete invasion, whereas no change in the number of melanized parasites would indicate an effect at a later stage. Analysis of the results as described above (REML variance component analysis and Chi-square goodness-of-fit test) revealed a significant 2.7-fold decrease (P<0.001) of the mean melanized parasite density in *ApoII/I* kd mosquitoes (Figure 3A, 3B and Table S3), as well as a marked decrease (23.9%; P<0.01) of the infection prevalence (Table S3). These results were independently confirmed by meta-analysis of the standardized mean difference of the various experimental replicates (Figure S2B). Living oocysts were not detected and complete disruption of egg development was again observed (data not shown).

The results presented above (Figure 2) clearly disconnect the functions of the *A. gambiae* ApoII/I and ApoIII in the response to *Plasmodium* infection and in mosquito egg development. We examined this disconnection further using the L3-5 mosquito infection assay. Indeed, in contrast to *ApoII/I*, depletion of *ApoIII* in such mosquitoes increased drastically (2.7-fold, P<0.001) rather than decreased the density of melanized parasites in the midgut (Figure 3A, 3B and Figure S2; and Table S3), further indicating that the two genes have different functions. Since we did not detect a similar significant increase of live oocyst density in ApoIII-depleted Yaoundé mosquitoes (see Figure 2), we hypothesize a role for ApoIII in parasite melanization. This is corroborated by our recent unpublished data showing an inhibitory effect of ApoIII on the prophenoloxidase activation cascade.

The *ApoII/I* kd data in L3-5 and Yaoundé mosquitoes suggest that this molecule facilitates *P. berghei* survival either at the pre-ookinete or at the ookinete stage. Another plausible explanation of the L3-5 phenotype could be that ApoII/I is also directly involved with the melanization cascade in these mosquitoes, but as a positive regulator. We tested and excluded this hypothesis on the basis of genetic epistasis experiments that examined the effect of *ApoII/I* silencing in another genetic background, *CTL4* kd Yaoundé mosquitoes (Figure 3C and S2C and Table S3). *CTL4* kd leads to direct melanization and subsequent killing of midgut-invading ookinetes [6]. Concurrent silencing of *CTL4* and *ApoII/I* led to a 2-fold drop in the total parasite load (melanized and not), as compared to the load in *CTL4* kd alone (P<0.05; Figure 3C). Therefore, the *ApoII/I* kd apparently affects survival of parasites before they reach the basal subepithelial space where they become melanized. No significant difference was observed in the proportion of melanized ookinetes to live oocysts between *CTL4* kd and *CTL4/ApoII/I* dkd (double knockdown) mosquitoes, further suggesting that ApoII/I is not involved in the melanization reaction per se.

Interestingly, the total parasite numbers in *CTL4* kd mosquitoes (Table S3) were significantly higher than in their controls (2.7-fold, P<0.001). Rather than suggesting a novel role for *CTL4*, we favor the interpretation that a large number of dead ookinetes are melanized in the absence of the melanization inhibitor CTL4, instead of undergoing lysis.

### *Plasmodium* attrition in *ApoII/I* kd and *WASP* kd mosquitoes

**Figure 4:**
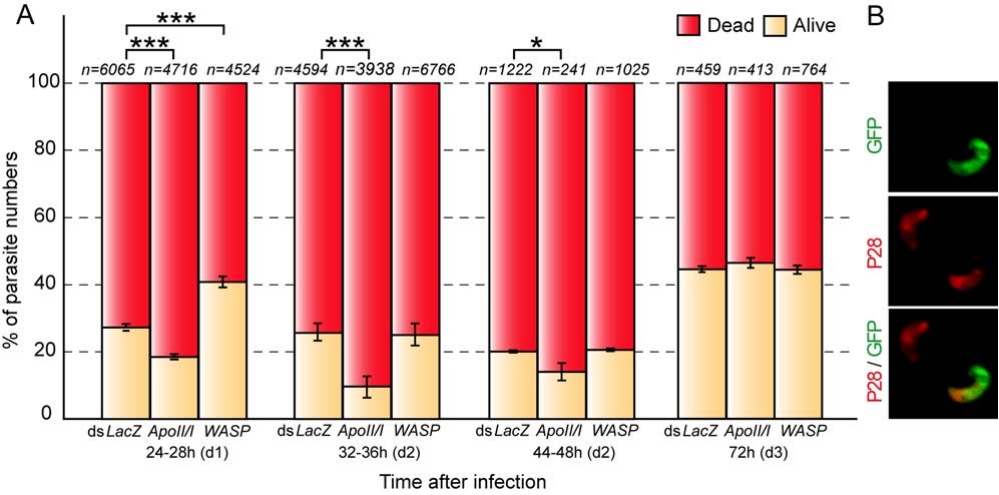
Effects of *ApoII/I* and *WASP* silencing on *P. berghei* killing during the ookinete-to-oocyst transition. (A) The graph shows temporal changes at the indicated hours in GFP fluorescing and anti-P28 antibody stained only parasites. Live ookinetes (yellow) display both GFP fluorescence and anti-P28 antibody staining, while parasites showing only anti-P28 antibody staining are regarded as dead (red). Note that relative to the *dsLacZ* control, *WASP* kd and *ApoII/I* kd mosquitoes show significantly lower and higher fractions of dead parasites, respectively. Error bars represent standard error in the pooled data set of at least three independent biological replicates. The total number (*n*) of assessed parasites is presented above each bar. The probability (*P*) of each gene kd being similar to the control ds*LacZ*-treated mosquitoes was calculated with the chi-square goodness-of-fit test for the observed vs. expected values. Asterisks indicate statistically significant effects of gene kd (*P*<0.05; *, *P*<0.001; ***). Brackets show the data set comparisons. (B) Representative images from three-dimensional projections of confocal sections of midguts infected with GFP fluorescent parasites (green) dissected at 24–28 h and stained with anti-P28 antibody (red). The ookinete at the top left corner is dead and displays only anti-P28 staining, whereas the ookinete at the bottom right corner is alive and exhibits both GFP and P28 expression.

Previous studies have shown that dead ookinetes of the *Pb*GFP_CON_ transgenic parasite line rapidly loose GFP fluorescence, but continue to display the ookinete surface protein P28 until the early oocyst stage [1],[6]. P28 antibody staining and confocal microscopy of infected midguts was used to assess the proportion of live to dead parasites in control (*LacZ* dsRNA-treated), *WASP* kd and *ApoII/I* kd Yaoundé mosquitoes, at various times after the infected bloodmeal (Figure 4). At day 1 only 17% of the ookinetes were alive (GFP and P28 positive) in the *ApoII/I* kd compared to 28% in the control mosquitoes. Chi-square goodness-of-fit test established the significance of this difference between observed and expected values (P<0.001, *x* ^2^ = 318.62). The effect was maximal at early day 2, 32–36 h (P<0.001, *x* ^2^ = 347.25) when only 10% of the parasites were alive in *ApoII/I kd* compared to 25% in control midguts. A less pronounced difference was observed at late day 2, 44–48 h (P<0.05, *x* ^2^ = 5.02), when 14% of the parasites (including the newly formed oocysts) were alive in *ApoII/I* kd compared to 20% in control midguts. No difference was observed at day 3. These results suggest that *ApoII/I* kd affects the ookinete during midgut invasion and also the early oocyst stages.

The kinetics were different in *WASP* kd mosquitoes, where dead ookinetes (GFP negative) were already 63% of the total on day 1 vs. 72% in controls (P<0.001; *x* ^2^ = 143.13); this effect disappeared at 32–36 h, day 2 and day 3. These data suggest that WASP acts at the level of midgut penetration by ookinetes.

### ApoII/I appear not to interact directly with midgut-invading ookinetes or oocysts

The *A. gambiae* ApoII/I precursor gene is expressed in the mosquito fat body. After cleavage of its 26 aa signal peptide, the protein is secreted into the hemolymph where it is proteolytically processed to release ApoII (688 aa) and ApoI (2618 aa) [25]. We raised monoclonal antibodies against two peptides (residues 59–70 and 3265–3276) targeting ApoII and ApoI, respectively. Western blot analysis showed that these antibodies recognize abundant hemolymph proteins of the respective size (Figure S3). The anti-ApoI antibody detected additional minor bands of lower molecular weight consistent with previous observations in *Manduca sexta,* that Apolipophorins are susceptible to proteolytic cleavage [20].

We used the antibodies to examine whether ApoI and/or ApoII bind to *P. berghei* ookinetes or oocysts in infected mosquito midguts. Immunostaining followed by confocal microscopy only detected a weak signal due to non-specific secondary antibody binding (data not shown).

In light of the complete disruption of mosquito egg development in the *ApoII/I* kd, we stained mosquito eggs with ApoI and ApoII antibodies at days 1, 2 and 3 after a bloodmeal. The staining detected both proteins in the apical area but not in the rest of the cytoplasm of the follicular epithelial cell layer (Figure S3), or within the oocyte and the nurse cells. The apical epithelial staining confirms that our antibodies can indeed recognize ApoII/I native proteins. Taken together, the immunostainings are consistent with a shuttling function of ApoII/I, a complex which is thought to transport lipids from the hemolymph to the follicular epithelium, where the lipid is internalized for storage in the egg while the lipoproteins are released back into the circulation.

## Discussion

In recent years, molecular interactions between mosquitoes and malaria parasites have received considerable attention, as vector mosquitoes represent a major bottleneck in the malaria transmission system. Such interactions have been studied mainly in laboratory models, and have identified several mosquito genes, often immune-related, which affect the infection outcome, positively or negatively. Studies involving the major vector of human malaria, *A. gambiae*, have been of special interest, but mostly utilized rodent parasites, or laboratory cultures of the human, *P. falciparum* parasite. In natural, sympatric parasitism systems, immune interactions may be shaped by selection acting on the vector, the parasite, or both and, as such, these effects may be specific to the parasite species. Indeed, the limited studies on interactions between geographically related *A. gambiae*/*P. falciparum* have suggested considerable differences between naturally interacting populations and laboratory models [11],[12],[26]. The present study begins to explore the significance of genes that do not belong to the classical immune repertoire, but have been implicated in local epithelial or systemic responses of *A. gambiae* to *P. berghei*.

Our study area was a rain forest locale with continuous malaria transmission and exposure peaking during rainy seasons, April to mid-June (when the study was conducted), or September to late November. To by-pass the daunting difficulties of direct experimentation under natural transmission conditions, we have adopted an approach where field isolates of *P. falciparum* are used to infect a geographically related laboratory colony (Yaoundé) of M molecular form *A. gambiae* such as 4ar/r. Although this colony was established in 1988 [21], it retains considerable genetic diversity, substantially higher than typically inbred laboratory strains of *A. gambiae*. Retention of genetic diversity may be due to the population size of this strain which has always been maintained at a high level, thus mitigating unintended selection processes.

While genetic divergence between the Yaoundé strain and local field collected mosquitoes is statistically significant, this divergence is limited compared to what is commonly observed between intraspecific populations and laboratory strains (data herein and in [22]). In conclusion, the Yaoundé strain is slightly diverged from the local population in the area of study, but it is still highly polymorphic and reasonably representative of the natural M form *A. gambiae* population. Moreover, recent data (A. Cohuet, unpublished data) have revealed that *P. falciparum* infection levels are highly similar between the Yaoundé strain and a new strain colonized some months ago also from local populations (N'gousso strain, I. Morlais, unpublished). Thus, it appears that the Yaoundé strain has not diverged significantly in respect to susceptibility to *P. falciparum* infection, despite absence of contact with *P. falciparum* for a number of generations.

Transcriptional responses of a mosquito gene to a parasite are considered indicative of a role during infection, although constitutively expressed genes may also be implicated in vector/parasite interactions. Previous studies identified drastic differences and limited similarities between mosquito transcriptional responses to *P. berghei* vs. laboratory [2] or field isolates of *P. falciparum* [12],[26]. These studies mostly focused on the mosquito immune system and differences were interpreted as due to *A. gambiae*/*P. falciparum* co-adaptation. Guided by our detailed microarray study of midgut responses to *P. berghei* [5], we focused here on five genes, of which only one belongs to the mosquito immune repertoire (*KIN1*). We report that all but *ApoIII* are upregulated during midgut invasion by both *P. berghei* and *P. falciparum* field isolates. Although some induction is evident in the respective controls at day-1 after a bloodmeal with non-infectious parasites, upregulation is stronger in the presence of ookinetes. Both Apolipophorin genes are also induced in the carcass in a parasite-independent manner, a response consistent with their putative function in lipid transport.

ApoI and II are cleaved from a common ApoII/I precursor and are integral components of the mosquito lipophorin, a versatile and reusable shuttle system for lipid transport [27]. Lipophorin transports dietary lipids from the gut via hemolymph to storage sites such as the fat body, muscles, ovaries and other tissues [28]. It also binds and delivers lipid-linked morphogens and glycophosphatidylinositol (GPI)-linked proteins to target cells of developing embryos [29]. These functions are consistent with the apical localization of ApoII/I protein at the follicular epithelium, and the prevention of ovarian maturation by ApoII/I depletion. ApoIII, the third polypeptide in lipophorin, is thought to counterbalance the increased hydrophobicity due to lipid binding and thus may stabilize lipophorin particles. However, it is evidently dispensable for lipid transport to the ovaries, as silencing this gene has no effect on follicle maturation.

In contrast, ApoII/I is an exemplar protein that benefits both the mosquito vector and the parasite: its depletion compromises egg production, ookinete invasion of the midgut and early oocyst development. The importance of this gene is highlighted by its requirement for development of the avian [30] as well as human and rodent [5] *Plasmodium* parasites. Indeed, ApoII/I is the first mosquito gene with a confirmed positive role in development of geographically-related *P. falciparum* field isolates. It validates the concept of mosquito agonists for human as well as rodent and avian parasites, and may prove to be a universal *Plasmodium* agonist.

Future studies are required to establish the mechanism whereby this important lipoprotein promotes ookinete migration and development. Immunostaining did not detect direct binding to the parasite. An attractive hypothesis is that ApoII/I sequesters lipids from the hemolymph and releases them to the developing oocyst where sporogonic proliferation and massive membrane formation occur. Indeed, in *ApoII/I* kd mosquitoes some early oocysts appear to be arrested in development and may be targeted for destruction. This documented positive effect of ApoII/I on oocysts does not explain its earlier effect on ookinete invasion. ApoII/I probably has multiple functions. It may help rescue and release ookinetes as a side effect of lipid mobilization from the midgut epithelium, where suggestive massive droplets appear at the basal side where ookinetes emerge [31]. Thus depletion of ApoII/I might trap ookinetes in the toxic environment of the invaded midgut. ApoII/I is also immune-induced in *Drosophila* hemolymph [32], detected in clotting assays [33], and present in immune-activated haemocytes [34]. Furthermore, in the fat body of *Ae. aegypti*, *ApoII/I* is regulated by the Toll/Rel1 immune pathway [30]. Thus, it may also be involved in systemic non-classical immunity.

ApoIII strongly resembles the N-terminal domain of human Apolipoprotein E which is involved in lipid transport, lipopolysaccharide detoxification, phagocytosis and pattern recognition [20]. Although ApoIII is a known partner of ApoII/I in the insect lipophorin, its depletion does not affect *Plasmodium* development in susceptible mosquitoes, but does increase drastically the density of melanized *P. berghei* parasites in the L3-5 refractory strain. These data together with our recent unpublished data showing increased prophenoloxidase activity in ApoIII-depleted mosquito hemolymph suggest that ApoIII may interfere with the melanization reaction itself. It is possible that a number of killed but not melanized parasites in the L3-5 mosquitoes are melanized after depletion of the putative melanization inhibitor ApoIII. This hypothesis is consistent with a report that ApoIII in *Galleria mellonella* dampens activation of the prophenoloxidase cascade by *Bacillus subtilis* lipoteichoic acid [35], although another study detected an opposite effect [36].

In contrast to ApoII/I, WASP is a parasite antagonist, as its local induction in the midgut reduces mosquito infection by *P. berghei* and more so by *P. falciparum*. Similarly, ApoII/I is induced more strongly in *P. falciparum* infected midguts, but protects *P. berghei* better. The levels of induction of WASP and ApoII/I do not directly correlate with their functional potency. Whether such discrepancies reflect co-adaptation of naturally interacting species or other evolutionary processes remains to be determined.

WASP is thought to play a key role in actin cytoskeleton rearrangements in epithelial cells. Our previous *in vivo* imaging analysis of midgut invasion by *P. berghei* revealed extensive actin-based motility of the damaged epithelium and an actin-rich structure surrounding ookinetes as they exit the epithelial cell layer [37]. A similar fibrillar organelle-free structure was observed previously around *P. gallinaceum* ookinetes in a refractory, lytic strain of *A. gambiae* [38]. Depletion of positive regulators of actin polymerization, such as WASP, increases *P. berghei* density, whereas depletion of negative regulators decreases the density; therefore we proposed that this actin-rich structure which we named “parasite hood” might be a defense reaction against invading parasites [5],[17]. Recently another study renamed this structure “organelle-free actin zone” and reported that it is required for the clearance of dead parasites [39]. However, this conclusion cannot explain our observed differences in live parasite density that follow silencing of actin cytoskeleton regulators. The formation, regulation and role(s) of this actin-based structure require further investigation. Although *WASP* silencing has a similar effect on the *P. falciparum* infection load, no conclusive evidence has been reported to date as to whether *P. falciparum* is also associated with a hood or causes damage to the invaded epithelium similar to that reported for *P. berghei* [16].

*P. falciparum* is the deadliest human parasite. Its association with *A. gambiae* exacts a devastating toll in Africa. Through several thousand years of *A. gambiae*/*P. falciparum* co-evolution, the parasite apparently adapted to reduce the burden on the vector by limiting infection intensity [13],[40], while securing successful transmission to humans. Presumably, the vector also adapted to reduce infection loads thus avoiding the fitness cost of immune system activation [13],[41]. Indeed, recent studies showed that several *A. gambiae* genes act as positive or negative regulators of immune reactions against *P. berghei* (a parasite that this mosquito has never encountered in nature), but do not affect sympatric *P. falciparum* infections [12],[42]. During co-evolution, parasites may also have developed specific mechanisms to modulate activation levels of the vector immune system. These diverse possibilities merit further analysis.

The present study, encompassing both high and low infection densities, demonstrates that genes implicated in local epithelial or systemic (but non-classical immune) responses can have similar effects but different activity levels against two different parasite species. These reactions may have some margin for adjustment in sympatric vector/parasite combination, but adjustment is probably limited for responses that are essential for the vector, e.g. to reconstitute the damaged midgut epithelium after parasite invasion (WASP) or to support reproduction (ApoII/I). Therefore, unlike widely adjustable immune responses that require investigation in natural interacting species, essential vector responses may be studied conveniently in model vector/parasite systems. Moreover, such conserved, robust and not widely adjustable interactions may be ideal for development of novel malaria control strategies.

## Materials and Methods

### Mosquito populations and strains

The Yaoundé colony was originally established at OCEAC, Cameroon, from a population of *A. gambiae s.s.* caught in a quarter of Yaoundé and adapted to feeding on parafilm membrane feeders [21]. This colony belongs to the M molecular and Forest chromosomal forms (standard chromosomal arrangement). Yaoundé, 4ar/r [23] and refractory L3-5 [11] mosquitoes were cultured in the insectary using standard methods. For the SNP analysis, *A. gambiae* larvae were collected in Simbock (03°51′N, 11°30′E), a South Cameroon village near Yaoundé and reared in the insectary until adult emergence.

### DNA isolation and sequencing

DNA was isolated from legs of 8 adult females from each of the laboratory colonies (Yaoundé and 4ar/r) and from the field collected M form *A. gambiae*, as described by Morlais et al. [43]. The M molecular form mosquitoes were distinguished from S form mosquitoes by a PCR assay [44]. PCR primer pairs for 10 immune related genes [45] were designed using Primer3 (http://frodo.wi.mit.edu/cgi-bin/primer3/primer3_www.cgi). The PCR reactions and PCR primers are presented in the Protocol S1 and in Table S4. Both DNA strands of PCR products were sequenced using an the Applied Biosystems 3730 sequencer, assembled and verified using SeqScape (Applied Biosystems).

### SNP data analysis

Sequence alignments were performed using ClustalW in MEGA 3.1 [46]. Polymorphism analyses and molecular population genetic test statistics were calculated using DnaSP 4.10 [47]. The nucleotide diversity within each population was estimated as the average pairwise nucleotide difference per site (π). Divergence between the wild populations and laboratory strains was assessed by sequence-based *F* statistics (Fst), analogous to Wright *F* statistics [48], which was calculated according to [49]; the net genetic distance was measured using D_a_ [50].

### Parasitological survey

Parasites were detected by microscopic examination of Giemsa-stained thick films of blood taken from volunteers by finger pricking. Gametocyte density was estimated assuming a standard number of 8000 WBC/µl of blood and by counting visible gametocytes against 1000 WBC. Children with asexual parasitaemia exceeding 1000 parasites/µl were treated with amodiaquine and artesunate combination according to national guidelines. Asymptomatic gametocyte-positive children were enrolled as volunteers, following procedures approved by the Cameroonian and WHO ethical review committees.

### *A. gambiae* infections with *P. falciparum*

For the expression profiling experiments, two groups of mosquitoes from the same rearing culture were used per replicate: the test group fed on blood donated by a gametocyte carrier, whereas the control group fed on the same blood which was previously incubated at 42–43°C for 12 min under constant shaking at 500 rpm for gametocyte inactivation. For the gene silencing experiments, we also used two groups of freshly emerged female mosquitoes per gene and per replicate, also separated randomly from the same rearing culture in small containers. The first group was subjected to silencing of the examined gene whereas the second control group was injected with dsRNA of the *LacZ* gene. In both cases, the two groups were housed under the same microclimate and treated identically, both before and after the blood feeding.

Mosquitoes were allowed to feed via a membrane on blood donated by *P. falciparum* gametocyte carries. To eliminate transmission blocking immunity factors, the carrier serum was replaced by non-immune AB serum [51]. Blood samples (700 µl each) were transferred into pre-warmed (37°C) artificial membrane feeders and exposed to mosquitoes that were previously starved for 12 hours, according to standard procedures [21]. To determine the levels of infection, mosquito midguts were dissected 8–10 days post blood feeding and stained with 2% mercurochrome before microscopic examination.

### *A. gambiae* infections with *P. berghei*

Three *P. berghei* clones, the gametocyte-producer ANKA 15cy1A (2.34), the non-gametocyte-producer ANKA 15cy1A (2.33) and the GFP-expressing PbGFP*_CON_* strain [52], were used for the various mosquito infections. Similar to what is described in the previous paragraph, two mosquito groups were used in each of the expression profiling and gene silencing experiments. For expression profiling, the test group fed on mice infected with the ANKA 2.34 strain and the control group fed on mice infected with the non-gametocyte producing ANKA 2.33 strain. In the gene silencing experiments, the two groups were treated as described for the *P. falciparum* infections and in [5], and fed on mice infected with the PbGFP*_CON_* strain. The infections were performed as described [53].

### RNAi gene silencing

Production of dsRNA and mosquito gene silencing was performed as described previously [5],[24].

### Quantitative real time RT-PCR (qRT-PCR)

QRT-PCR expression profile analysis of mosquito genes were performed as described previously [5]. Briefly, total RNA was extracted using the TRIZOL reagent (Invitrogen) from 30–50 mosquitoes for expression profiling and from 10 adult female mosquitoes to determine the gene silencing efficiency. For the expression profile analysis, two to four independent biological replicates were performed, which used different batches of mosquitoes fed on different blood sources (different gametocyte carriers for the *P. falciparum* infections and different infected mice for the *P. berghei* infections). The results of each biological replicate were the average of two technical replicates, in which the same RNA samples were processed in duplicate in the same qRT-PCR plate. The *A. gambiae* S7 ribosomal gene was used as an internal control to normalize the amount of RNA between the various samples (i.e. between kd and control mosquitoes). For assessment of the gene silencing efficiency, dsRNA-injected mosquitoes (control and kd) were collected before blood feeding. Gene-specific primers for *ApoII/I*, *WASP*, *CATHB* and *KIN1* were used for qRT-PCR and dsRNA as previously described by [5]. The qRT-PCR and dsRNA primers for *ApoIII* were: forward, 5′-GCCGTGCAGGGAAGCTT-3′; reverse, 5′-ATCTTGTCCTTGATGCTCATGA-3′ for qRT-PCR and forward, 5′-TAATACGACTCACTATAGGGTCCAGTCGATCATGAGCATCA-3′; reverse, 5′-TAATACGACTCACTATAGGGAGCTTCTTGAGCGCGTCCT-3′ for dsRNA production.

### Antibody production

Peptides corresponding to the *A. gambiae* ApoI N-terminal sequence (^+^HN-CGNYAQQKTPKDKKQ-COO^−^) and ApoII C-terminal sequence (^+^HN- CGLQQSDKENKQT-COO^−^) were synthesized (ams biotechnology) and used by the EMBL Monoclonal Antibody Facility to immunize mice and thereby generate hybridoma cell-lines.

### Western blot analysis

Hemolymph was obtained by clipping the proboscis of female *A. gambiae* mosquitoes and collecting a hemolymph droplet in a pipette tip filled with reducing SDS loading buffer (0.25M Tris pH6.8, 40% Glycerol, 8% SDS, 8% *β*-mercaptoethanol). Proteins were resolved by discontinuous SDS-PAGE, with 5% stacking and 8% resolving gel, and subsequently transferred to Hybond-P membranes (Amersham Biosciences) in a Trans-Blot SD Semi-Dry Transfer Cell (BioRad) blotting chamber. Unspecific antibody binding was reduced by blocking the membranes overnight (o/n) at 4°C in blocking buffer (1% Tween and 3% Milk powder in PBS). Supernatants of hybridoma cell lines containing primary antibodies were diluted 1∶100 in blocking buffer, and membranes were incubated o/n at 4°C. Anti-mouse IgG conjugated to horseradish peroxidase (Promega) was used as a secondary antibody at 1∶15000 dilution in blocking buffer; incubation was performed for 1–3 h at RT. Blots were developed using Western Lightning Chemiluminescence Reagent Plus Kit (PerkinElmer Life Sciences).

### Immunohistochemistry and confocal microscopy

Mosquito midgut epithelia and ovaries were dissected in ice-cold PBS and fixed in 1×PBS pH 7.2, 4% Formaldehyde, 1 µmol EGTA, 2 µmol MgSO_4_. After washing (PBS at RT for 3×15 min) and blocking (midguts: 1× PBS pH7, 0.1% Triton X-100, 1% BSA; ovaries: 1× PBS pH7, 0.2% Saponin, 1% BSA), the tissues were incubated with monoclonal antibodies against ApoI (1∶100) and ApoII (1∶100) followed by incubation with Alexa-647-conjugated goat anti-mouse antibodies (Molecular Probes) at 1∶1,500 for 90 min at RT. Tissues were washed and mounted on microscope slides using VECTASHIELD Mounting Medium with DAPI (Vector Labs). For negative controls, tissues were incubated in blocking buffer without antibodies. For parasite killing assays, infected midguts were incubated with a Cy-3-tagged monoclonal antibody against *P. berghei* P28 at 1∶750 (a kind gift of R. E. Sinden). Mounted tissues were observed on a Leica SP2 or Leica SP5 confocal microscope or Leica DMR microscope, respectively. Image processing was performed with the ImageJ v1.36b software.

### Data analysis

The oocyst density data were log-transformed [log_10_ (*n*+1)] so that their distribution resembles a normal distribution. A linear model was used to examine the relationship between the oocyst densities and gametocyte densities, in which the oocyst density was the response variable and the gametocyte density was the explanatory variable. The correlation between the gametocyte and mosquito infection prevalence was investigated by fitting a logistic regression model, where the absence or presence of infection was used as the response variable and the gametocyte density as the explanatory variable. In the RNAi experiments, the oocyst density data were analyzed by the REML variance components analysis by fitting a mixed effect model. The kd-control status was treated as a fixed effect and we introduced a random effect for the biological replicate. For each dataset a combined *P*-value is reported for the fixed effects. The difference in the infection prevalence between kd and their control mosquitoes were analyzed using the Chi-square goodness-of-fit test where the observed values were fitted to expected values. Similarly, the same test was used to examine the difference in the proportion of live/dead parasites between gene kd and their control mosquitoes in the killing assays. All the above statistical tests were performed using the GenStat software.

Meta-analysis of the standardized mean difference of the various biological replicates in the gene silencing experiments, and calculation of the total standardized mean difference with 95% Confidence Interval were performed by using the Comprehensive Meta-analysis software (Biostat, version 2). This analysis uses two models, fixed and random, and the total standardized mean difference is given both for the fixed and the random effect model.

### Accession numbers

Ensembl accession numbers for reported genes and proteins are as follows**:** ApoII/I (AGAP001826-PA, AGAP001826); WASP (AGAP001081-PA, AGAP001081); KIN1 (AGAP005888-PA, AGAP005888); CATHB (AGAP007684-PA and AGAP007684-PB, AGAP007684); ApoIII (ENSANGESTG00000005174); CTL4 (AGAP005335-PA, AGAP005335); CEC2 *(*AGAP000692-PA, AGAP000692); SCRB10 (AGAP000016-PA, AGAP000016); STAT2 (AGAP000099-PA, AGAP000099); SRPN11 (AGAP001377-PA, AGAP001377); GNBPB2 (AGAP002729-PA, AGAP002729); PPO9 (AGAP004978-PA, AGAP004978); LRIM1 (AGAP060348-PA, AGAP060348); TEP15 (AGAP008364-PA, AGAP008364); TEP4 (AGAP010812-PA, AGAP010812); TOLL10 (AGAP011187-PA, AGAP011187).

## Supporting Information

Protocol S1 (0.05 MB DOC)

Table S1 Summary of *P. falciparum* parasitological survey in Mfou, Cameroon
(0.04 MB DOC)

Table S2 Genetic diversity in natural population from Cameroon, Yaoundé and 4ar/r strains of *A. gambiae* M molecular form, and genetic distance between natural population and strains
(0.05 MB DOC)

Table S3 Effect of *A. gambiae* gene silencing on *P. falciparum* and *P. berghei* development
(0.07 MB DOC)

Table S4 PCR primers for SNP analysis
(0.10 MB DOC)

Figure S1 Efficiency of gene silencing
(3.68 MB TIF)

Figure S2 Forest plots of meta-analysis for standardized mean difference of gene silencing effect on parasite development
(1.38 MB TIF)

Figure S3 Expression and in situ localization of ApoI and ApoII in *A. gambiae*
(1.09 MB TIF)
