# Supplementary material for: Conserved Mosquito/Parasite Interactions Affect Development of Plasmodium falciparum in Africa
Source: PLoS Pathog. 2008 May 16;4(5):e1000069. doi: 10.1371/journal.ppat.1000069 (PMC2373770; doi:10.1371/journal.ppat.1000069)
Supplement: Protocol S1 — (0.05 MB DOC) [file ppat.1000069.s001.doc]

**Protocol S1**

Conserved Mosquito/Parasite Interactions Affect Development of *Plasmodium falciparum* in Africa

**Antonio M. Mendes,^1^* Timm Schlegelmilch,^1^* Anna Cohuet,^2^ Parfait Awono-Ambene,^3^ Maria De Iorio,^4^ Didier Fontenille,^2^ Isabelle Morlais,^3^ George K. Christophides,^1^ Fotis C. Kafatos,^1^† and Dina Vlachou^1^†**

**Supporting materials and methods**

PCR reactions for SNP analysis

PCR reactions were performed in 50 µl solution containing 20 pmol of each primer, each dNTP at 0.2 mM, 2.5 mM MgCl_2_, 10 mM Tris-HCl (pH 8.3), 50 mM potassium chloride (KCl), 2 units of Taq polymerase and approximately 10 ng of template DNA. Amplification conditions included an initial 5 min 94°C denaturation, followed by 12 cycles at 94°C for 30s, 65°C for 30s, with a decrease of one degree per cycle, and finally 72°C for 1 min 30s. This step was followed by 25 cycles of 94°C for 30s, 56°C for 30s, and 72°C for 1 min 30s, and a final 72°C, 10 min extension step. Both DNA strands were sequenced using an Applied Biosystems 3730 sequencer. Sequences were assembled and verified using SeqScape (Applied Biosystems).

**Supporting figure legends**

**Figure S1**. Efficiency of gene silencing

(A) Residual RNA levels in whole mosquitoes were estimated by qRT-PCR. For calculation of RNA abundance in kd compared to control ds*LacZ*-treated mosquitoes (100%), data from at least three representative biological replicates were included. Bars represent standard deviation. The average efficiency of gene silencing was 65.0% (±7.5) for *ApoII/I*, 62.3% (±5.7) for *WASP*, 51.3% (±12.4) for *CATHB*, 73.5% (±2.8) for *KIN1* and 81.4% (±5.8) for *ApoIII*.

(B) ApoII/I knockdown by RNAi is efficient in A. gambiae Yaoundé strain mosquitoes. An immunoblot of hemolymph proteins isolated from 20 mosquitoes treated with dsApoII/I or dsLacZ as a control was probed with mouse anti-ApoI and anti-ApoII antibodies, and rabbit anti-Srpn 3 antibody (1:750, kindly provided by K. Michel) for loading control.

(C) Residual protein levels in mosquito hemolymph. Blots were scanned with an Epson 2450 PHOTO scanner. Protein band intensities and their respective background were determined with the Photoshop CS2 software. Median intensities were used to determine the percentage of ApoI and ApoII depletion compare to control mosquitoes (100%). The average reduction at protein level was 77% (±6) and 73% (±4) for ApoI and ApoII, respectively. Data from three biological replicates were included. Bars represent standard deviation.

**Figure S2**. Forest plots of meta-analysis for standardized mean difference of gene silencing effect on parasite development

The standardized mean difference for the various biological replicates (red lines), as well as the total standardized mean difference (blue lines) for each dataset are presented with 95% Confidence Interval (CI). The total standardized mean difference is given both for the fixed effect model (upper blue line) and the random effect model (lower blue line). Note that the effect of gene kd on parasite development is significant when *P* values for both models are significant, thus demonstrating no heterogeneity of the data between replicates. The total number of midguts (N) in each dataset is shown.

(A) Effect of RNAi gene silencing of *ApoII/I*, *WASP*, *CATHB*, *KIN1* and *APOIII* on *P. falciparum* field isolates (first column) or *P. berghei* (second column).

(B) Effect of *ApoII/I* and *ApoIII* silencing on *P. berghei* in the *A. gambiae* L3-5 mosquitoes.

(C) Analysis of the results of *ApoII/I* and *CTL4* genetic epistasis experiments.

**Figure S3**. Expression and *in situ* localization of ApoI and ApoII in *A. gambiae*

(A) Immunoblot of ApoI and ApoII proteins in mosquito hemolymph. Proteins were sampled 24 h post infection with *P. berghei* and then analyzed by 8% SDS-Page. Apolipophorins were detected with mouse monoclonal anti-ApoI (left lane) and anti-ApoII (right lane) peptide antibodies. Numbers indicate protein size scale in kDa. A band at 220 kDa represents full length ApoI protein; lower molecular weight bands may represent products of ApoI proteolytic cleavage. The major band at 75 kDa in the right lane correlates with the expected molecular weight of ApoII.

(B) In situ localization of ApoII/I in ovarian follicles. Anti-ApoI (red; top panel) and anti-ApoII (red; middle panel) antibodies were used for immunofluorescence confocal imaging. Cell nuclei were stained with DAPI (blue). Single confocal sections are shown in each panel. ApoII and ApoI proteins are distributed along the follicular cell epithelium at day-1 post bloodmeal. No red signal is detected in control ovaries stained only with secondary antibodies and DAPI (bottom panel). OC; oocyte compartments, FCE; follicular cell epithelium, FCN; follicular cell nuclei.
