# Supplementary material for: Conserved Mosquito/Parasite Interactions Affect Development of Plasmodium falciparum in Africa
Source: PLoS Pathog. 2008 May 16;4(5):e1000069. doi: 10.1371/journal.ppat.1000069 (PMC2373770; doi:10.1371/journal.ppat.1000069)
Supplement: Table S1 — Summary of P. falciparum parasitological survey in Mfou, Cameroon (0.04 MB DOC) [file ppat.1000069.s002.doc]

| **Table S1.** Summary of *P. falciparum* parasitological survey in Mfou, Cameroon | | | | | | | |
| --- | --- | --- | --- | --- | --- | --- | --- |
|  |  |  | ABS | |  | Prevalence (%) | |
| Season | School | Screened | (+) | (-) | Gametocytes | Total | Gametocytes |
| May-05 | Annex Public | 714 | 378 | 336 | 43 | 52.9 | 6.0 |
|  | Bilingual | 178 | 117 | 61 | 15 | 65.7 | 8.4 |
|  | Mekoumba | 132 | 56 | 76 | 4 | 42.4 | 3.0 |
|  | Subtotal | 1024 | 551 | 473 | 62 | 53.8 | 6.1 |
| May-06 | Annex Public GrII | 764 | 402 | 462 | 43 | 106.0 | 11.5 |
|  | Bilingual | 180 | 65 | 115 | 10 | 36.1 | 5.6 |
|  | Mekoumba | 196 | 126 | 70 | 5 | 64.3 | 2.6 |
|  | Public | 654 | 239 | 415 | 28 | 71.5 | 8.3 |
|  | Nkilzok | 177 | 112 | 65 | 13 | 63.3 | 7.3 |
|  | Adoum | 86 | 52 | 34 | 10 | 60.5 | 11.6 |
|  | Subtotal | 2057 | 996 | 1061 | 109 | 48.4 | 5.3 |
| Total |  | 3081 | 1547 | 1534 | 171 | 51.0 | 5.9 |
