# Supplementary material for: Conserved Mosquito/Parasite Interactions Affect Development of Plasmodium falciparum in Africa
Source: PLoS Pathog. 2008 May 16;4(5):e1000069. doi: 10.1371/journal.ppat.1000069 (PMC2373770; doi:10.1371/journal.ppat.1000069)
Supplement: Table S2 — Genetic diversity in natural population from Cameroon, Yaoundé and 4ar/r strains of A. gambiae M molecular form, and genetic distance between natural population and strains (0.05 MB DOC) [file ppat.1000069.s003.doc]

| **Table S2**. Genetic diversity in natural population from Cameroon, Yaoundé and 4ar/r strains of *A. gambiae* M molecular form, and genetic distance between natural population and strains. | | | | | | | | | | | | | |
| --- | --- | --- | --- | --- | --- | --- | --- | --- | --- | --- | --- | --- | --- |
|  |  |  | Diversity | | |  | Genetic distance | | | | | | |
|  |  |  |  |  |  |  | Yaoundé strain / natural population | | |  | 4ar/r strain / natural population | | |
| Gene | Chr  location | Sequence size (bp) | π  natural | π Yaoundé | π  4ar/r |  | Fst | Da | *P* value |  | Fst | Da | *P* value |
| CEC2 | X | 180 | 0.013 | 0 | 0 |  | 0.0667 | 0.0001 | 0.493 |  | 0.8963 | 0.0056 | 0 |
| SCRB10 | X | 607 | 0.0009 | 0.0002 | 0 |  | 0.027 | 0.0002 | 0.0143 |  | 0.4 | 0.0003 | 0.0082 |
| STAT2 | X | 434 | 0.0017 | 0 | 0 |  | 0.022 | 0 | 0.282 |  | 0.0222 | 0 | 0.3349 |
| SRPN11 | 2R | 474 | 0.0156 | 0.0176 | 0 |  | -0.14 | -0.002 | 0.276 |  | 0.408 | 0.0053 | 0.0028 |
| GNBPB2 | 2R | 599 | 0.0202 | 0.019 | 0.0024 |  | 0.158 | 0.0046 | 0.001 |  | 0.2044 | 0.0046 | 0.0449 |
| PPO9 | 2L | 636 | 0.0137 | 0.0045 | 0.0023 |  | 0.439 | 0.007 | 0.0518 |  | 0.3267 | 0.0051 | 0.0316 |
| LRIM1 | 2L | 546 | 0.004 | 0.0063 | 0 |  | 0.3121 | 0.0023 | 0.002 |  | 0.6667 | 0.004 | 0 |
| TEP15 | 3R | 535 | 0.0116 | 0.0142 | 0 |  | 0.0583 | 0.0008 | 0.3353 |  | 0.7944 | 0.022 | 0.0055 |
| TEP4 | 3L | 765 | 0.005 | 0.0077 | 0.0006 |  | 0.1018 | 0.0007 | 0.2745 |  | 0.4254 | 0.003 | 0.0651 |
| TOLL10 | 3L | 578 | 0.0088 | 0.0113 | 0 |  | 0.1571 | 0.0018 | 0.0374 |  | 0.5528 | 0.0054 | 0.0091 |
| Overall |  | 535.4 | 0.0095 | 0.0081 | 0.0005 |  | 0.1202 | 0.0016 | 0* |  | 0.4697 | 0.0055 | 0* |
| π : nucleotide diversity for each population and each locus. Arithmetic mean was used to measure diversity overall locus. Fst: genetic differentiation between natural population and strains. Da: net genetic distance between natural population and strains. Arithmetic mean was used to measure genetic distances overall locus. *: Fisher’s test was used to estimate significance overall locus. | | | | | | | | | | | | | |
