# Supplementary material for: Conserved Mosquito/Parasite Interactions Affect Development of Plasmodium falciparum in Africa
Source: PLoS Pathog. 2008 May 16;4(5):e1000069. doi: 10.1371/journal.ppat.1000069 (PMC2373770; doi:10.1371/journal.ppat.1000069)
Supplement: Table S3 — Effect of A. gambiae gene silencing on P. falciparum and P. berghei development (0.07 MB DOC) [file ppat.1000069.s004.doc]

| **Table S3**. Effect of *A. gambiae* gene silencing on *P. falciparum* and *P. berghei* development | | | | | | | | |
| --- | --- | --- | --- | --- | --- | --- | --- | --- |
|  |  |  |  | Parasite density | |  | *P* |  |
| DsRNA | # of exp | # of midguts | Prevalence (%) | Arithmetic mean | Geometric mean (±SE) | Parasite range | Mixed Effects Model RELM variance components analysis | Fold difference |
| ***P. falciparum field isolates / A. gambiae* (Yaoundé strain)** | | | | | | | | |
| Apo II/I | 4 | 143 | 69.2 | 4.6 | 2.37 (±0.01) | 0-23 | <0.001*** | -1.6 |
| LacZ |  | 143 | 75.5 | 7.6 | 3.79 (±0.17) | 0-58 |  |  |
| WASP | 5 | 89 | 80.9‡ | 21.9 | 7.63 (±0.47) | 0-193 | <0.001*** | 3.7 |
| LacZ |  | 89 | 44.9 | 10.5 | 2.04 (±0.47) | 0-120 |  |  |
| CATHB | 3 | 42 | 47.9 | 2.6 | 1.16 (±0.24) | 0-24 | 0.357 | -0.7 |
| LacZ |  | 42 | 54.7 | 4.1 | 1.59 (±0.31) | 0-32 |  |  |
| KIN1 | 4 | 117 | 53.8 | 3.7 | 1.57 (±0.04) | 0-33 | 0.1 | 1.6 |
| LacZ |  | 117 | 41.9 | 2.4 | 0.95 (±0.04) | 0-32 |  |  |
| ApoIII | 5 | 120 | 50.0 | 4.0 | 1.44 (±0.18) | 0-53 | 0.185 | 1.4 |
| LacZ |  | 120 | 40.0 | 2.8 | 1.03 (±0.16) | 0-32 |  |  |
| ***P. berghei* / *A. gambiae* (Yaoundé strain)** | | | | | | | | |
| ApoII/I | 7 | 116 | 61.2‡ | 16.6 | 4.09 (±0.38) | 0-136 | <0.001*** | -4.7 |
| LacZ |  | 116 | 85.3 | 65.7 | 19.79 (±0.41) | 0-550 |  |  |
| WASP | 5 | 73 | 89.0 | 44.2 | 16.27 (±0.46) | 0-326 | <0.001*** | 2.5 |
| LacZ |  | 73 | 73.0 | 20.7 | 6.41 (±0.46) | 0-124 |  |  |
| CATHB | 5 | 122 | 78.7 | 38.0 | 9.55 (±0.45) | 0-295 | 0.82 | 1.1 |
| LacZ |  | 122 | 77.1 | 47.4 | 9.14 (±0.50) | 0-450 |  |  |
| KIN1 | 5 | 58 | 74.1 | 28.2 | 9.82 (±0.23) | 0-163 | 0.301 | 1.3 |
| LacZ |  | 58 | 79.3 | 18.8 | 7.30 (±0.20) | 0-91 |  |  |
| ApoIII | 6 | 94 | 84.0 | 24.3 | 8.19 (±0.38) | 0-132 | 0.833 | -1.0 |
| LacZ |  | 94 | 81.9 | 33.4 | 8.42 (±0.41) | 0-360 |  |  |
| ***P. berghei* / *A. gambiae* (L3-5 refractory strain)** | | | | | | | | |
| ApoII/I | 4 | 54 | 64.8‡ | 21.1 | 6.37 (±0.81) | 0-167 | <0.001*** | -2.7 |
| LacZ |  | 54 | 85.2 | 47.4 | 17.15 (±0.68) | 0-287 |  |  |
| ApoIII | 5 | 76 | 82.9 | 95.6 | 38.00(±0.62) | 0-330 | <0.001*** | 2.7 |
| LacZ |  | 76 | 82.9 | 32.4 | 13.99 (±0.41) | 0-171 |  |  |
| **Genetic epistasis** | | | | Total | Total |  |  |  |
| ApoII/I | 4 | 61 | 62.3 | 8.7 | 2.74 (±0.36) | 0-67 | <0.001*** | -3.7 |
| CTL4 |  | 61 | 90.2 | 73.0 | 27.32 (±0.56) | 0-355 | <0.001*** | 2.7 |
| CTL4/ApoII/I |  | 61 | 86.9 | 37.7 | 13.85 (±0.49) | 0-155 | 0.291 | 1.4 |
| LacZ |  | 61 | 86.9 | 26.8/ | 10.06 (±0.53) | 20-250 |  |  |
| The table is divided into four datasets, each including results from gene kd and control (LacZ dsRNA-treatment) experimental groups. Within each group, each gene kd has its own control, except in the Genetic epistasis dataset, where all genes used the same control. Kd and their control mosquitoes were fed with blood from the same gametocyte carrier or fed on the same infected mouse. The total number of midguts is indicated in the third column. Prevalence shows the percentage of midguts with at least one oocyst (‡; statistically significant difference of infection prevalence between kd and control mosquitoes as analyzed by Chi-square test of association). Midguts with zero oocysts were also considered for calculation of the arithmetic and geometric means of parasite densities (number per midgut). In the Genetic epistasis dataset, mean densities of live, melanized parasites and their sum (total) are presented. Asterisks indicate statistically significant effects of gene kds (*P*<0.005; ***) as determined by the Residual Maximum Likelihood (REML) variance components analysis. Fold differences between oocyst densities of kd and control mosquitoes were computed using the geometric means. | | | | | | | | |
