# Supplementary material for: Conserved Mosquito/Parasite Interactions Affect Development of Plasmodium falciparum in Africa
Source: PLoS Pathog. 2008 May 16;4(5):e1000069. doi: 10.1371/journal.ppat.1000069 (PMC2373770; doi:10.1371/journal.ppat.1000069)
Supplement: Table S4 — PCR primers for SNP analysis (0.10 MB DOC) [file ppat.1000069.s005.doc]

| **Table S4.** PCR primers for SNP analysis | | | | |
| --- | --- | --- | --- | --- |
| Gene | Gene ID | Primer Name | Accession numbers | Primer Sequence |
| CEC2 | AGAP000692 | CEC2-F | AM774761-AM774785 | 5’-CTATAAAATGCGCCCGGTTG-3’ |
|  |  | CEC2-R | AM900849-AM900862 | 5’-CCGAGGTTTGAACTGCTCAC-3’ |
| SCRB10 | AGAP000016 | SCRB10-F | AM774672-AM774696 | 5’-AGTGTGGCATGTCAGGATGA-3’ |
|  |  | SCRB10-R | AM900904-AM900917 | 5’-ACCGAGCAAAACGAAACAGT-3’ |
| STAT2 | AGAP000099 | STAT2-F | AM774697-AM774721 | 5’-GAGAAGGCGTTCAAAACGAA-3’ |
|  |  | STAT2-R | AM900926-AM900938 | 5’-GTGTTCGGGAAGCTGTTCAT-3’ |
| SRPN11 | AGAP001377 | SRPN11-F | AM774811-AM774830 | 5’-CTCCTGTCAACGATTTAAAGGC-3’ |
|  |  | SRPN11-R | AM900918-AM900925 | 5’-ATGGTTTCGGAAGCAAGTCTT-3’ |
| GNBPB2 | AGAP002729 | GNBPB2-F | AM774987-AM775011 | 5’-CGTTCAGCTTCAGGTATGGAA-3’ |
|  |  | GNBPB2-R | AM900863-AM900876 | 5’-GCACGTAGTCAATCTGTAGCGA-3’ |
| PPO9 | AGAP004978 | PPO9-F | AM775311-AM775335 | 5’-CCCGAACGCTATCAACGTAT-3’ |
|  |  | PPO9-R | AM900891-AM900903 | 5’-TCAGCAGCTTCGGGAAGTAT-3’ |
| LRIM1 | AGAP060348 | LRIM1-F | AM775468-AM775492 | 5’-ACGGTGCAATACACGAGATAAAG-3’ |
|  |  | LRIM1-R | AM900877-AM900890 | 5’-CCTTAGCGCTTTCTCGATCA-3’ |
| TEP15 | AGAP008364 | TEP15-F | AM775617-AM775641 | 5’-CGACCATACAGAACCTGGACT-3’C |
|  |  | TEP15-R | AM900953-AM900965 | 5’-GAATCACTGTCGGTCGTTGC-3’ |
| TEP4 | AGAP010812 | TEP4-F | AM775907-AM775931 | 5’-ACCGCGGCAAAGTACATATC-3’ |
|  |  | TEP4-R | AM900939-AM900952 | 5’-GTTTGCGTTGGTTACCGATT-3’ |
| TOLL10 | AGAP011187 | TOLL10-F | AM776029-AM776052 | 5’-ACGCGTCCACGATCTATCTG-3’ |
|  |  | TOLL10-R | AM900966-AM900978 | 5’-GCACATAGCCACCACCAATC-3’ |
