# Supplementary figures and images for: Conserved Mosquito/Parasite Interactions Affect Development of Plasmodium falciparum in Africa
Source: PLoS Pathog. 2008 May 16;4(5):e1000069. doi: 10.1371/journal.ppat.1000069 (PMC2373770; doi:10.1371/journal.ppat.1000069)

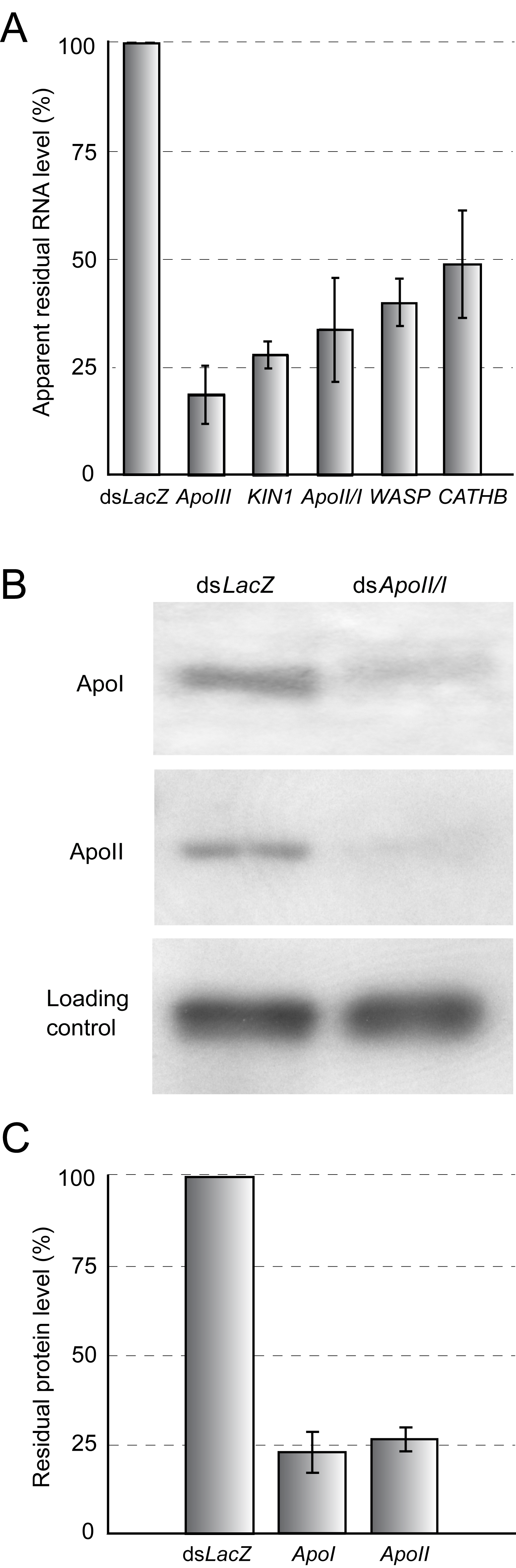

Supplement: Figure S1 — Efficiency of gene silencing (3.68 MB TIF) [file ppat.1000069.s006.tif]

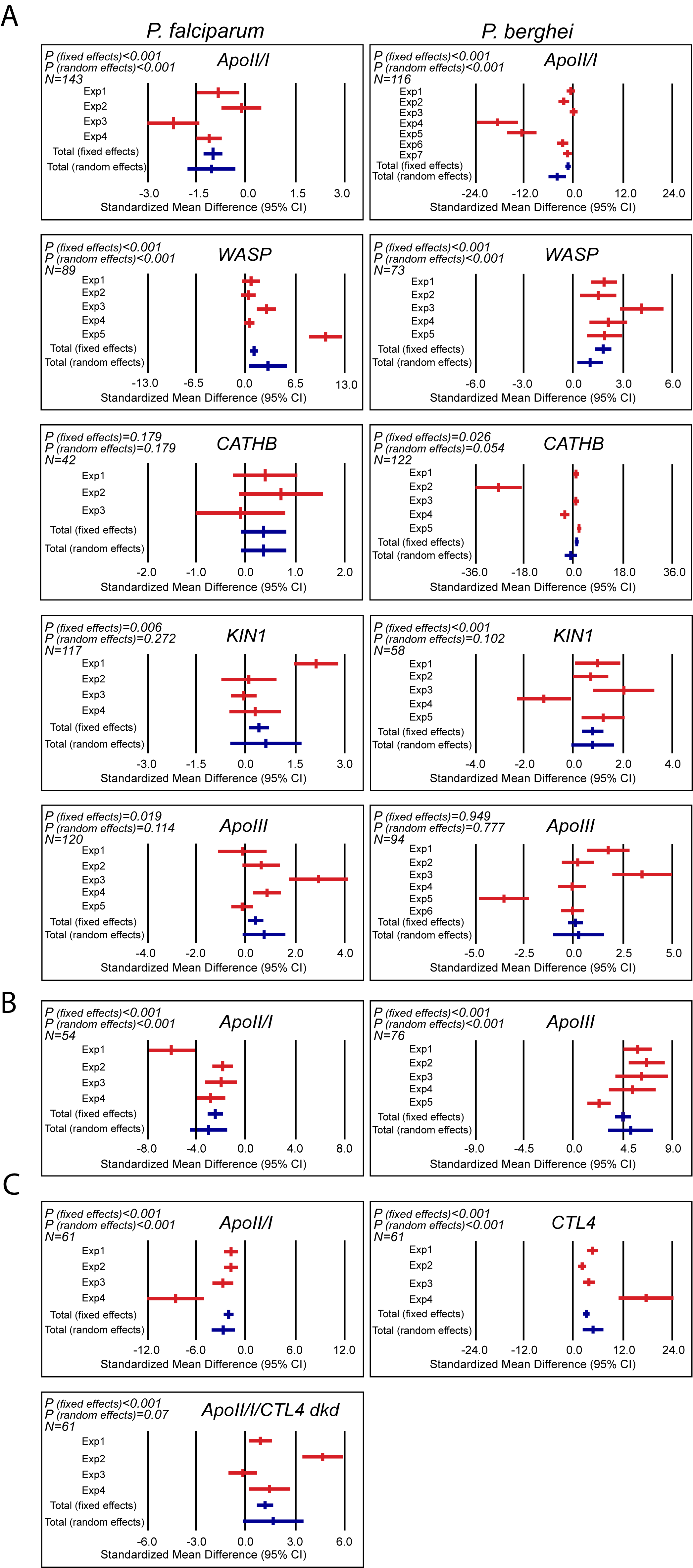

Supplement: Figure S2 — Forest plots of meta-analysis for standardized mean difference of gene silencing effect on parasite development (1.38 MB TIF) [file ppat.1000069.s007.tif]

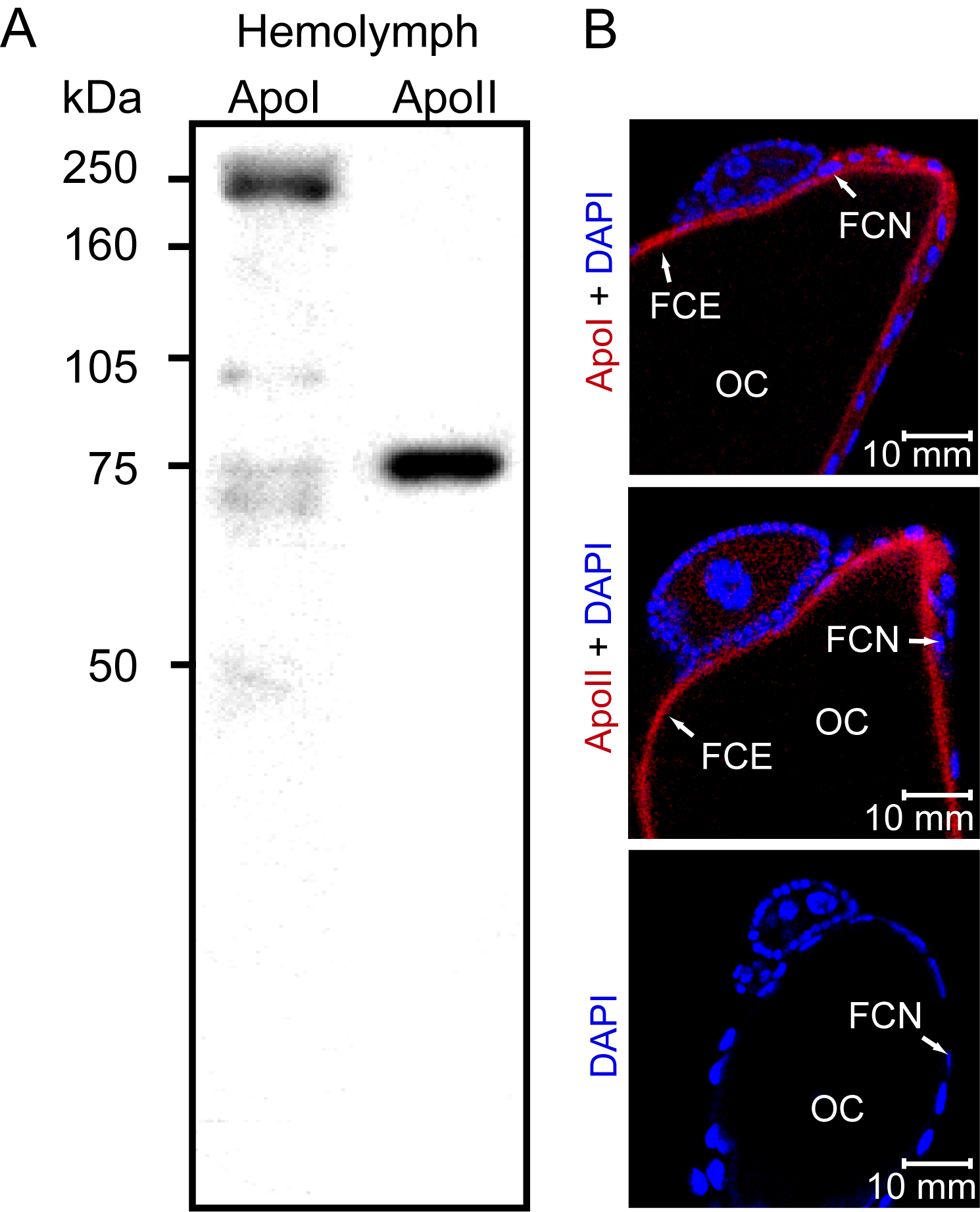

Supplement: Figure S3 — Expression and in situ localization of ApoI and ApoII in A. gambiae (1.09 MB TIF) [file ppat.1000069.s008.tif]
